# Blood haemoglobin measurement as a predictive indicator for the progression of HIV/AIDS in resource-limited setting

**DOI:** 10.1186/1423-0127-16-102

**Published:** 2009-11-18

**Authors:** Christian Obirikorang, Francis A Yeboah

**Affiliations:** 1 Department of Molecular Medicine, School of Medical Sciences, College of Health Sciences, Kwame Nkrumah University of Science and Technology (KNUST), Kumasi, Ghana; 2 University Hospital, Laboratory Department, KNUST, Kumasi, Ghana

## Abstract

**Background:**

Anaemia is a frequent complication of infection with the human immunodeficiency virus (HIV) and may have multiple causes. The objective of this study was to find out if blood haemoglobin measurement could be used as an indicator for the progression of HIV/AIDS in resource-limited setting.

**Methods:**

Two hundred and twenty-eight (228) consented People Living with HIV/AIDS (PLWHAs) who were placed in three groups according to their CD4 counts were used in the study. The three groups were those with CD4 counts (1) ≥ 500 mm-^3^; (2) 200-499 mm-^3^; and (3) *<*200 mm-^3^. One hundred (100) sex, age-matched and healthy HIV-seronegative individuals were used as control subjects. Blood haemoglobin, blood haematocrit, Red cell indices which included Mean Cell Volume, Mean Cell Haemoglobin Concentration and Mean Cell Haemoglobin and CD4 count were analysed in all subjects.

**Results:**

The mean blood haemoglobin concentrations in those with CD4 counts *<*200 mm-^3^, 200-499 mm-^3 ^and ≥ 500 mm-^3 ^(8.83 ± 0.22 g/dl, 10.03 ± 0.31 g/dl and 11.3 ± 0.44 g/dl respectively) were significantly lower when compared with the control group (14.29 ± 0.77 g/dl) (*p *< 0.0001). The mean blood haematocrit levels in those with CD4 counts *<*200 mm-^3^, 200-499 mm-^3 ^and ≥ 500 mm-^3 ^(23.53 ± 0.85%, 28.28 ± 0.77% and 33.54 ± 1.35% respectively) were also significantly lower when compared with the control group (41.15 ± 2.15%) (*p *< 0.0001). The red cell indices were also lower in the subjects when compared with the control group. Using the Pearson's correlation, there was a significant and positive correlation between the blood haemoglobin level and their CD4 counts *(*r^2 ^= 0.1755; *p < 0.0001)*.

**Conclusion:**

Anaemia in People Living with HIV/AIDS, if persistent, is associated with substantially decreased survival. From our analysis, there was a decrease in the blood haemoglobin, levels as the HIV infection progressed and our findings are consistent with those of other studies of anaemia as a prognostic factor in HIV infection. Haemoglobin levels could be measured easily where resources for more sophisticated laboratory markers such as viral load or even CD4 lymphocyte count are not available given that measurement of the CD4 lymphocyte count requires flow cytometry, an expensive technique unavailable in many developing countries. Regular measurements could help to determine which patients are at greatest risk of disease progression, allowing these patients to be identified for closer monitoring or therapeutic intervention.

## Background

HIV infection is the chief cause of morbidity and mortality among adults and children, especially in sub-Saharan Africa. At the end of 2007, 40 million persons worldwide were living with HIV or AIDS [1]. Anaemia is a frequent complication that occurs in 20-80% of HIV-infected persons and is associated with faster disease progression and mortality [2]. This makes it more common than thrombocytopenia or leucopenia in patients with AIDS [3]. Therefore, interventions to prevent anaemia may lead to improved health and survival potential of HIV-infected persons [4].

HIV infection may lead to anemia in many ways: changes in cytokine production with subsequent effects on hematopoiesis [5,6] decreased erythropoietin concentrations [7] opportunistic infectious agents, such as *Mycobacterium avium *complex [8] and parvovirus B- [9] administration of chemotherapeutic agents such as zidovudine, ganciclovir, [10] and trimethoprimsulfamethoxazole [11] and myelophthisis caused by cancers such as lymphosarcoma. Other mechanisms for HIV-associated anaemia, although uncommon, include vitamin B12 deficiency [12] and the autoimmune destruction of red blood cells[13]. Anaemia has been associated with progression to AIDS and shorter survival times[14] for HIV-infected patients.

While CD4 count and HIV-RNA are the gold standard markers for disease monitoring in PLWHAs, when measurement of these parameters is not possible surrogate markers become important. Markers investigated for their utility as simple markers for disease progression in resource-limited settings include delayed type hypersensitivity responses (DTH), total lymphocyte count (TLC), haemoglobin and body mass index (BMI) [15].

Haemoglobin levels reflect rapidity of disease progression rates and independently predict prognosis across demographically diverse cohorts [16,17]. Rates of haemoglobin decrease also correlate with falling CD4 counts [18,19]. There have been suggestions that increases in haemoglobin are predictive of treatment success [20]. While racial variation in normal haemoglobin ranges and the side effects of antiretroviral agents such as zidovudine on the HIV infected bone marrow must be taken into account [21], monitoring haemoglobin levels shows utility in predicting disease progression both before and following HAART initiation.

The aim of this study was, therefore, to determine if blood haemoglobin measurement could be used as an indicator for the progression of HIV/AIDS in limited resource-limited settings. We sought to compare the change in haemoglobin levels in the patients as the disease progressed to a Control group who were one hundred (100) sex- and age-matched healthy, HIV-seronegative individuals. In addition, we sought to investigate the relationship between haemoglobin and CD4 lymphocyte count in the HIV individuals.

## Methods

This study was a prospective case control comparative study which was conducted at one of the established centers providing Anti Retroviral Therapy (ART) and is located at Cape Coast, the capital of the Central region of Ghana. The study was conducted between August 2007 and May 2008. Blood samples were taken from the subjects before the initiation of ART. This study was approved by the Committee on Human Research, Publications and Ethics (CHRPE), School of Medical Sciences, Kwame Nkrumah University of Science & Technology (KNUST), Kumasi. All patients enrolling in the study completed a written informed consent form in accordance with the Helsinki Declaration. After obtaining consent, demographic questionnaires were completed. Blood was drawn for CD4 cell count, blood haemoglobin, blood haematocrit and Red cell indices. The normal reference range for blood haemoglobin was 12.0-18.0 g/dl and that of blood haematocrit was 37-55%. The definition of anaemia chosen for this study was therefore blood haemoglobin less than 12 g/dl and blood haematocrit less than 37%. We chose this cut-off for defining anaemia to exclude hereditary causes for mild anaemia, such as thalassemia trait, and to allow for use of a single cut-off that would clearly exclude normal haemoglobin concentrations for both men and women.

In all one hundred and fifty (150) confirmed People Living with HIV/AIDS (PLWHAs) were included in the study. PLWHAs were placed in three (3) groups according to the Centers for Disease Control and Prevention Criteria (CDC) classification system that emphasizes the importance of CD4+ T lymphocyte testing in clinical management of HIV-infected persons. The system is based on three ranges of CD4 counts (1) ≥ 500 mm-^3^; (2) 200-499 mm-^3^; and (3) *<*200 mm-^3 ^. Control subjects were one hundred (100) sex- and age-matched healthy (39.4 ± 13.4 years), HIV-seronegative individuals.

The blood haemoglobin, blood haematocrit and Red cell indices were determined by automated blood analyzer (CELL-DYN 1800, Abbott Laboratories Diagnostics Division, USA). A modified Methemoglobin method was used for the colorimetric determination of hemoglobin. A portion of the lysed, diluted sample from the WBC Mixing Chamber was used for HGB measurement. A low-energy Light-Emitting Diode (LED) was used as the light source. The LED shone through the HGB flow cell and a 540 nm narrow-bandwidth filter onto a photo detector. The HGB concentration was directly proportional to the absorbency of the sample.

The CD4 T lymphocytes count was determined using the Becton Dickinson (BD) FASCount system (Becton, Dickinson and Company, Califonia, USA). The BD FASCount system used flow cytometry for the quantification of the CD4 T Lymphocytes. Flow cytometry uses the principles of light scattering, light excitation, and emission of fluorochrome molecules to generate specific multi-parameter data from particles and cells in the size range of 0.5 um to 40 um diameter. Flow cytometry is the recognized gold standard for CD4 testing, and is used to stage HIV/AIDS, guide treatment decisions for HIV-infected persons, and evaluate effectiveness of therapy.

### Statistical Analysis

The OUTLIERS preliminary test for detection of error values was initially applied for statistical analysis. The results were given as mean ± Standard error of mean (SEM). Correlations were evaluated using the Pearson's correlation test. For all statistical comparisons, the level of significance was set at *p *< 0.05. All data analysis in this research was done using GraphPad Prism for Windows version 4.02 (GraphPad Software, San Diego, CA, USA).

## Results

Demographic characteristics of patients used in this study are shown below in table 1. There were no significant difference between the ages of the test subjects and the control. There were however significant differences in the CD4 count between the control group and those with CD4 count <200 (*p *< 0.0001) and 200-499 (*p *< 0.0001).

**Table 1:** Demographic characteristic of the Subjects and Controls.

Parameter	Control	Total Subjects	CD4>500	CD4 200-500	CD4<200
Age, (years)	38.76 ± 1.73	37.10 ± 0.84	38.72 ± 2.58	37.43 ± 1.57	36.68 ± 1.08
Males, (n)	72	157	22	47	88
Females, (n)	28	71	21	16	34
CD4 count, (mm^-3^)	1045.45 ± 77.53	226.0+21.17***	823.1 ± 60.75	310.70 ± 14.44***	84.30 ± 77.53***
HIV serotype 1, (n)	-	138	22	34	82
HIV serotype 2, (n)	-	1	-	-	1
HIV serotype 1 and 2,(n)	-	89	20	27	42

The values are expressed as mean ± SEM

**Figure 1:**
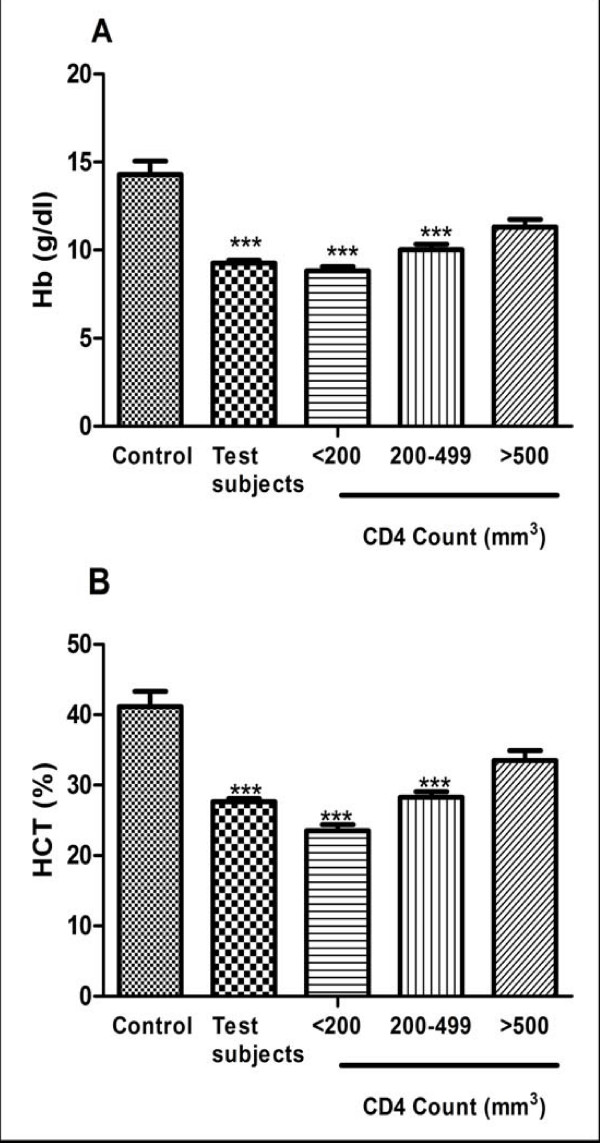
**CDC staging of CD4 counts on (A) Blood haemoglobin and (B) Blood haematocrit in HIV patients and control group**. The results are presented as means ± SEM. ***P ≤ 0.0001, compared to respective control (one way ANOVA followed by Bonferroni's *post hoc*). (Hb: haemoglobin, HCT: haematocrit, CDC: Centre for Disease Control).

The mean blood haemoglobin level of the test subjects were 11.3 ± 0.44 g/dl, 10.03 ± 0.31 g/dl and 8.83 ± 0.22 g/dl for CD4 ≥ 500 mm^-3^, 200-499 mm^-3 ^and ≤ 200 mm^-3 ^respectively and that of the control group was 14.29 ± 0.77 g/dl as shown in figure 1A. The mean blood haematocrit level of the test subjects were 33.54 ± 1.35%, 28.28 ± 0.77% and 23.53 ± 0.85% for CD4 ≥ 500 mm^-3^, 200-499 mm^-3 ^and ≤ 200 mm^-3 ^respectively and that of the control group was41.15 ± 2.15% as shown in figure 1B.

Blood haemoglobin and haematocrit levels decreased suggesting increased risk of developing anaemia as the HIV infection progressed. At a cut-off of 12.0 g/dl, 82.40% of the test subjects were found to be anaemic with 4.1% of the control group also being anaemic.

One-way ANOVA revealed a significant difference between the test subjects and the control group (F_3_, _228 _= 13.24; *p *< 0.0001). A further post-test analysis using the Bonferroni's Multiple Comparison Test revealed that there was a significant difference between the control group and the subjects with CD4 count <200 and CD4 count between 200 and 499 (*p *< 0.0001). There was however no significant difference between the control group and those with CD4 count greater than 500 (*p *= 0.13). There was also a significant different between the subjects with CD4 count greater than 500 and those with CD4 count less than 200 (*p *< 0.001).

**Figure 2:**
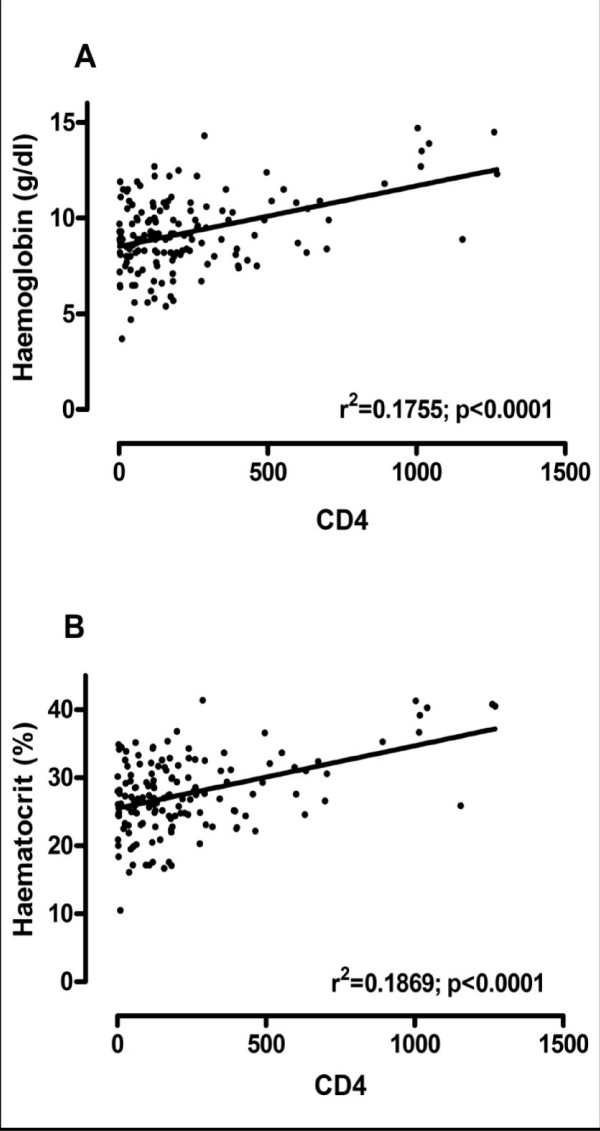
**Linear correlation between CD4 count and blood (A) Haemoglobin and (B) Haematocrit, in patients with HIV infection**. (r = correlation coefficient).

Using the Pearson's correlation, there was a significant and positive correlation (r^2 ^= 0.1755; p < 0.0001) between the blood haemoglobin level and their respective CD4 counts as shown in figure 2A. Thus as the blood CD4 count of the subjects decreased there was a decrease in their respective blood haemoglobin levels. Blood haematocrit also had a similar trend as the blood haemoglobin. A correlation analysis between the CD4 count of the test subjects and their respective haematocrit levels revealed a significant and positive correlation (r^2 ^= 0.1869; *p *< 0.0001) as shown in figure 2B.

**Figure 3:**
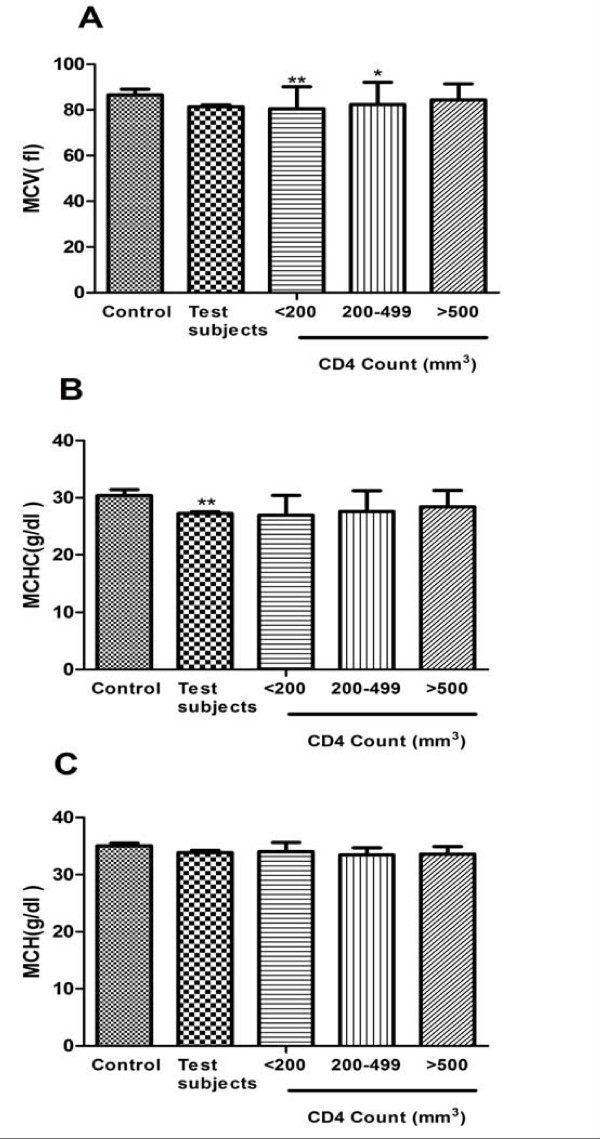
**CDC staging of CD4 counts on (A) MCV (B) MCHC and (C) MCH in HIV patients and control group**. The results are presented as means ± SEM. **P ≤ 0.001, *P ≤ 0.05 compared to respective control (one way ANOVA followed by Bonferroni's *post hoc*). (MCV: Mean Cell Volume, MCH: Mean Cell Haemoglobin MCHC: Mean Cell Haemoglobin concentration, CDC: Centre for Disease Control).

The mean values for MCV, MCH and MCHC are shown in figure 3. There were significant difference between the MCV of the control group and those with CD4<200 (*p *< 0.001) and 200-499 (*p *< 0.05). There were however no significant difference between the MCH of the control group and the various groups of the test subjects.

## Discussion

The incidence of anemia was strongly and consistently associated with the progression of HIV disease as measured by CD4 count (figure 3). This association is most likely explained by the increasing viral burden as HIV disease progresses, which could cause anemia by increased cytokine mediated myelosuppression. Alternatively, anemia may be a surrogate marker for some aspect of disease progression not captured by controlling for CD4 count and clinical AIDS diagnosis. Our study results are consistent with previous studies which showed that low haemoglobin levels increase the risk of AIDS [22,23] increase the risk of death in patients with AIDS [24-26] and increase the risk of death among patients with advanced immunodeficiency[27,28], both in cross-sectional [29] and longitudinal studies [22]. Several staging systems have suggested that important information can be provided by haemoglobin levels [27,28,30]. Significant and positive correlation was found when blood haemoglobin level was compared with their respective CD4 count (Figure 2) of the subject group. Our results also show that haemoglobin levels provide prognostic information independent of that provided by the CD4 lymphocyte count.

Although the cut-offs we used to define anaemia differ from those in a paper by [31], this results agree well with this recent study, which used the latest measure of haemoglobin and CD4 lymphocyte count in a similar way to our analysis [32]. From the Red cell indices analysis, most of the test subjects had low MCV and MCHC as compared to the control group. Our findings are consistent with those of [33] who found that the majority of test subjects presented with microcytic hypochromic anaemia which is common in most chronic diseases (figure 3).

Most of our data was collected before common use of the new highly active antiretroviral therapies. Before the introduction of HAART, investigators reported that the annual loss of CD4 lymphocyte counts was 40-80 × 10^6 ^cells/l [34-37]. Measurement of the loss of haemoglobin during HIV infection has not previously been reported. It was interesting to note that the change in haemoglobin in our study was gradual and a continual process. There have been considerable changes in the availability of antiretroviral regimens and in the way they are combined in the EuroSIDA study [38]. The pathogenesis of HIV-associated anaemia is unclear and is likely to be multifactorial in nature [39,40]. Possible causes are bleeding (gastrointestinal malignancy/severe infection), insufficient dietary intake (vitamins such as cobalamin and folate, iron, and general malnutrition), haemolytic anaemia (i.e. malignancies, infections, splenomegaly, and immune dysfunction), and changes in erythropoietin synthesis and/or bone marrow suppression. Suppression of the bone marrow in HIV-infected patients may be initiated in several ways. These include an action of HIV itself (infection of the progenitor of the red blood cell synthesis in late-stage disease), direct pathogenic involvement of the bone marrow (malignant lymphoma, atypical mycobacteriosis), prophylactic or therapeutic treatment against opportunistic diseases or malignancies (i.e. sulphamethoxazole, ganciclovir, methotrexate, adriamycin) or simply non-specific effects relative to chronic or acute infections.

Haemoglobin is both easy and inexpensive to measure and it was measured before the initiation of ART. Clearly patients who developed severe anaemia before the initiation of ART were at an increased risk of death. Monitoring haemoglobin levels could be used to alert clinicians to those patients who require more regular clinical follow-up or who may need treatment for their anaemia. Moore et al, (1998) found that treatment of anaemia with erythropoietin was associated with an improved prognosis possibly by allowing higher doses or prolonged use of drugs such as zidovudine and ganciclovir. In a recent cross-sectional study, haemoglobin measurement was shown to play an important role in the basic management of HIV disease in West Africa [41].

In conclusion, given its strong relationship with AIDS-defining illness and death, haemoglobin levels could be measured easily in PLWHAs where resources for more sophisticated laboratory markers such as viral load or even CD4 lymphocyte count are not available (given that measurement of the CD4 lymphocyte count requires flow cytometry, an expensive technique unavailable in many developing countries) [42]. Regular measurements of blood haemoglobin in PLWHAs in resource-setting could help to determine which HIV patients are at greatest risk of disease progression, allowing these patients to be identified for closer monitoring or therapeutic intervention.

## Competing interests

The authors declare that they have no competing interests.

## Authors' contributions

CO carried out the haematological analysis, CD4^+ ^counts, performed the statistical analysis and drafted the manuscript. FAY designed the study and its coordination and participated in the drafting of the manuscript. All authors read and approved the final manuscript.
